# Influence of Severity of Type and Timing of Retrospectively Reported Childhood Maltreatment on Female Amygdala and Hippocampal Volume

**DOI:** 10.1038/s41598-020-57490-0

**Published:** 2020-02-05

**Authors:** Julia I. Herzog, Janine Thome, Traute Demirakca, Georgia Koppe, Gabriele Ende, Stefanie Lis, Sophie Rausch, Kathlen Priebe, Meike Müller-Engelmann, Regina Steil, Martin Bohus, Christian Schmahl

**Affiliations:** 1 Department of Psychosomatic Medicine and Psychotherapy, Central Institute of Mental Health Mannheim, Medical Faculty Mannheim, Heidelberg University, J5, 68159 Mannheim, Germany; 2 Institute for Psychiatric and Psychosomatic Psychotherapy, Central Institute of Mental Health Mannheim, Medical Faculty Mannheim, Heidelberg University, J5, 68159 Mannheim, Germany; 3 Department of Neuroimaging, Central Institute of Mental Health Mannheim, Central Institute of Mental Health Mannheim, Medical Faculty Mannheim, Heidelberg University, J5, 68159 Mannheim, Germany; 4 Department of Theoretical Neuroscience, Central Institute of Mental Health Mannheim, Medical Faculty Mannheim, Heidelberg University, J5, 68159 Mannheim, Germany; 5 Department of Psychiatry and Psychotherapy, Central Institute of Mental Health Mannheim, Medical Faculty Mannheim, Heidelberg University, J5, 68159 Mannheim, Germany; 6 Department of Psychiatry, University of Western Ontario, 339 Windermere Rd., London, N6A 5A5 ON Canada; 7 Department of Psychology, Faculty of Life Sciences, Humboldt-University of Berlin, Unter den Linden 6, 10999 Berlin, Germany; 8 Department of Clinical Psychology and Intervention, Institute of Psychology, Goethe-University Frankfurt, Varrentrappstr. 40-42, 60486 Frankfurt am Main, Germany

**Keywords:** Stress and resilience, Post-traumatic stress disorder

## Abstract

Deleterious effects of adverse childhood experiences (ACE) on human brain volume are widely reported. First evidence points to differential effects of ACE on brain volume in terms of timing of ACE. Upcoming studies additionally point towards the impact of different types (i.e., neglect and abuse) of ACE in terms of timing. The current study aimed to investigate the correlation between retrospectively reported severity of type (i.e., the extent to which subjects were exposed to abuse and/or neglect, respectively) and timing of ACE on female brain volume in a sample of prolonged traumatized subjects. A female sample with ACE (N = 68) underwent structural magnetic resonance imaging and a structured interview exploring the severity of ACE from age 3 up to 17 using the “Maltreatment and Abuse Chronology of Exposure” (MACE). Random forest regression with conditional interference trees was applied to assess the impact of ACE severity as well as the severity of ACE type, (i.e. to what extent individuals were exposed to neglect and/or abuse) at certain ages on pre-defined regions of interest such as the amygdala, hippocampus, and anterior cingulate (ACC) volume. Analyses revealed differential type and timing-specific effects of ACE on stress sensitive brain structures: Amygdala and hippocampal volume were affected by ACE severity during a period covering preadolescence and early adolescence. Crucially, this effect was driven by the severity of neglect.

## Introduction

Adverse childhood experiences (ACE), i.e. sexual or physical abuse or neglect during childhood, are highly prevalent worldwide^1^. Particularly prolonged and repeated ACE constitutes a major risk factor for adult psychopathology^2^ such as major depression^3^, substance abuse^4^, personality disorders^5^, anxiety disorders, and posttraumatic stress disorder (PTSD)^6^. ACE is further linked to deleterious effects on neurocognitive functioning (i.e., working memory and inhibitory control), mirrored in significant functional and structural alterations in stress and emotion sensitive brain regions such as in the amygdala, hippocampus, as well as in the anterior cingulate cortex (ACC) (for reviews see^2,7^). It has been hypothesized that the latter brain regions are particularly vulnerable to the impact of ACE due to a high density of glucorticoid receptors; hence prolonged release of glucocorticoids is stated to cause damage, dendritic atrophy and neurogenesis suppression^7,8^. Yet, although the direction in terms of a reduction or enlargement of these regions varies across studies^7,9^, volumetric changes in stress and emotion associated brain regions are hypothesized to play a pivotal role in individual differences contributing to resilience or vulnerability in the aftermath of ACE, emphasizing the need to understand modulating factors of the relationship between brain volume and ACE.

Building on evidence from animal models, a novel conceptual framework has been proposed, which is increasingly highlighted in the field - deconstructing ACE into at least two underlying dimensions: *active* and *passive* maltreatment that may distinctly impact neural development^10^. Active maltreatment represents harmful experiences, challenging the physical integrity of the self, e.g. physical and sexual abuse^10^. Passive maltreatment consists of the absence of social and cognitive environmental input, which is necessary to fulfil the basic needs of a child, i.e. emotional and physical neglect^10^. Animal studies allow the development of experimental protocols in which animals are exposed to acute and/or chronic stress^11^. Hence, the cause–effect relationship between stress and its impact on the brain can be directly demonstrated. Experimental stressful ‘early-life’ manipulations in animals include e.g. separating the animal from its mother, modifying maternal behavior, or exposing the animal to synthetic glucocorticoids^11^. Animals exposed to stress pre- or postnatally show a wide range of changes in the brain’s neurochemical system, exhibit more learning errors and show alterations of the sensitivity of the HPA axis, thereby potentially altering the animal’s ability to regulate their emotional states^12^. Due to ethical issues, the cause–effect impact of stress on the brain cannot be studied in humans, and therefore human studies are correlational by nature, as the experience of abuse and neglect co-occur at extremely high rates in children and adolescents^13^. Consequently, finding individuals who only experienced one form of adversity would not only be difficult, but also would not accurately represent the population of children and adolescents exposed to ACE. Therefore, it seems reasonable to use the dimensions that are the severity of ACE types (i.e., abuse and neglect severity) within one sample, instead of separate categories (i.e., abuse vs. neglect). Studies focusing on passive maltreatment in subjects are predominantly those of early deprivation in institutionally reared children. In the English and Romanian Adoptees study, significantly smaller white and gray matter volume, as well as smaller volume of the left hippocampus, and larger volume in the right amygdala was observed for institutionalized adolescents adopted from Romania to the United Kingdom vs. never-institutionalized adoptees from the United Kingdom^14^; but see^15^. Moreover, a randomized clinical trial compared children, who remained in an institution in Bucharest to those that have been placed into high-quality foster care during early childhood and to non- institutionalized children^16,17^. Children exposed to institutional rearing showed decreased cortical gray matter and white matter compared to non-institutionalized children. However, children who were placed into foster care did not significantly differ in their white matter volume from those children reared in biological families^16^. Interestingly, no effects of institutionalization were found on subcortical regions such as the hippocampus and the amygdala. Studies focusing on active maltreatment, i.e. abuse, found evidence for a negative relation between (sexual) abuse and the size of the hippocampus^18^, visual cortex, as well as somatosensory cortex^18–20^. These results have to be interpreted with caution, due to the fact that abuse is usually accompanied by neglect^21^, making it difficult to study the relative contribution of abuse on development.

Only two studies directly compared childhood abuse and childhood deprivation so far. Everaerd *et al*. (2016) found reduced volume in the fusiform gyrus and middle occipital gyrus in individuals exposed to deprivation, compared to those exposed to abuse, while volume alterations in somatosensory areas (posterior precuneus, postcentral gyrus) were further modulated by gender^22^. Moreover, Teicher *et al*. (2018) showed that male hippocampal volume was associated with neglect, while female hippocampal volume was associated with abuse^23^.

The brain is shaped not only by the *type* of ACE encountered during development, but also by *timing*, referring to when ACE were experienced during brain development^7^. Neuronal plasticity is defined as the ability of the brain to adapt its structure and function in response to environmental demands, experiences and physiological changes^24,25^. Crucially, the human brain remains plastic throughout the whole life span^11,25^, whereby the degree of plasticity seems to be modulated by varying maturation trajectories of different brain regions^11,26^. In this light, one might has the possibility to detect the timing of the higher impact of ACE on neuroanatomical measurements. Recent investigations have therefore addressed the question, whether ACE has a distinct impact on brain morphology during specific time windows (for review see^27^:). Pechtel *et al*.^28^, showed that the right amygdala was affected by exposure to maltreatment at 10–11 years of age, and that only a modest degree of exposure was required to produce maximal hypertrophy^7^. Moreover, they found that right hippocampal volume appeared to be most affected to maltreatment at 7 and 14 years of age. A further study in women with a history of sexual abuse found evidence of a timing effect of ACE at 3–5 years of age as well as between 11–13 years of age on bilateral hippocampal volume^18^. Thus, recent studies have started to delineate timing effects of ACE pointing to a differential timing effect during preadolescence (about 9–12 years of age) and early adolescence (about 13 years of age) for the development of the amygdala and the hippocampus. The time of pre- and adolescence is characterized by marked changes in brain structure and function, as white and grey matter undergo complex changes, particularly in regions of the frontal cortex that are involved in higher-level cognitive processes^29^. Moreover, the limbic system (e.g., the hippocampus and the amygdala) undergo structural and functional maturation during this period^30,31^. Critically, hippocampal, amygdaloidal and cortical regions play a central role in stress reactivity due to their high density of corticosteroid receptors. These receptors detect glucocorticoid stress hormones and regulate the hypothalamic-pituitary-adrenal (HPA) axis^32^. As psychological and physiological stressors during pre- and adolescence have a negative impact on the HPA axis^33^, one may hypothesize that limbic and cortical regions might be especially vulnerable to stress during this time period^11,26^. The aim of the present study was to investigate the impact of retrospectively reported ACE on brain volume in relation to severity of type and timing in a sample of individuals exposed to repeated interpersonal trauma during childhood and adolescence. Severity of type was defined as the extent subjects were exposed to abuse and/or neglect, respectively. To achieve this aim, we first investigated the impact of retrospectively reported *global ACE severity*, *global abuse* and *neglect severity* on volumes of key stress and emotion associated brain structures, i.e. the amygdala, hippocampus, and ACC by pre-defined regions of interest (ROI). We decided to choose the amygdala, the hippocampus, and the ACC as regions of interest, since several studies from human and experimental animal studies demonstrated their sensitivity to early stressful events^21,26,34,35^. We only concentrated on these three ‘typical’ areas to avoid multiple testing, and thereby the risk of false positive results^36^. Second we aimed to replicate the findings of timing effects for the amygdala and hippocampus^18,28^ volume during which *time-specific ACE severity* has an impact on brain volume^18,28^. To our knowledge, studies have not so far demonstrated timing effects of the ACC in the context of ACE. Therefore we investigated timing effects within the ACC by exploratory analyses. Third, we were particularly interested if there is an interaction between the timing and the severity of type (*time-specific abuse and neglect severity*) on brain volume. Since the diagnosis of PTSD has also been related to alterations in the amygdala, hippocampus, and ACC (for reviews and meta-analyses see^37,38^ (but also^39,40^), we took the existence of a PTSD diagnosis in our analyses into account.

## Methods and Materials

### Sample

The sample consisted of 68 traumatized female participants who reported sexual or physical abuse during childhood and adolescence (inclusion criterion). Fourty-two participants fulfilled the primary diagnosis of posttraumatic stress disorder (PTSD), and 26 participants were free of any mental disorder throughout their life (trauma controls, TC^41^). Details on demographic and clinical characteristics, as well as maltreatment exposure history are reported in SI 1.1, SI 2.1 and Tables S1–S5. The study was approved by the Ethical Board II of Heidelberg University, Germany, and was conducted according to the Declaration of Helsinki at the Central Institute of Mental Health in Mannheim. Written informed consent was obtained from the participants after the procedures had been fully explained. All subjects received monetary remuneration for participation in the study.

### Maltreatment exposure

The time course and severity of reported exposure to traumatic events was assessed using an adapted version of the Maltreatment and Abuse Chronology of Exposure Interview (MACE^42,43^;). The inventory evaluates 10 types of ACE during each year of childhood up to age 17. Within the present investigation, ACE was quantified by a) an averaged MACE severity score indicating ACE across childhood and adolescence, (i.e. *global ACE severity*), and for each year of life, respectively (i.e. *time-specific ACE severity*)^42^. The scores range from 0 to 100. To address b) the conceptual framework of active and passive maltreatment^10,44^, we created two dimensions: Active maltreatment is represented by collapsing the subscales physical and sexual abuse (=*abuse*), while passive maltreatment is represented by collapsing the subscales physical and emotional neglect (=*neglect*). The scores have been averaged across childhood and adolescence, i.e. *global abuse severity*, and *global neglect severity*, as well as for each year of life, respectively i.e. *time-specific abuse severity*, and *time-specific neglect severity*. The neglect and abuse score ranges from 0 to 20 (for details see SI 1.2.1).

### Magnetic resonance imaging and image processing

For details on MRI procedure and image processing please see SI 1.2.2 and SI 1.2.3.

### Statistical analyses

Repeated measurement analysis of variance (rmANOVA) were applied to investigate differences in reported ACE, abuse, or neglect, respectively, across the life-span, i.e. 3 up to 17 years of age. Pearson correlations were conducted, to investigate the relationship between brain volume (amygdala, hippocampus, ACC) and maltreatment history (i.e., ACE, abuse, and neglect). If necessary, post-hoc comparisons were conducted and adjusted for multiple testing (Bonferroni). Statistical significance was set to *p* < 0.05. All analyses were performed using SPSS (version 23; SPSS Inc., USA). For further details on statistical analyses regarding the history of maltreatment, clinical, and socio-demographic characteristics see SI 1.3.

### Analysis of timing effects

To test the presence of timing effects in which exposure to ACE might be more strongly related to alterations in ROI brain volume, we applied random forest regression with conditional interference trees (‘cforest’ in R package ‘party’^45,46^;). Since *type and time-specific ACE severity* scores were highly intercorrelated (*p*-values < 0.030), we applied conditioned random forest regression to identify relevant predictors. This method is advantageous compared to conventional linear models in identifying important predictor variables, as random forest regression considers multicollinearity between predictor variables, while additionally handling a large number of predictor variables^46,47^. We ran the random forest regression with conditional interference trees for each ROI (GMV, age and TIV corrected, and z-transformed). Each random forest model consisted of 500 trees with randomly selected 4 variables available at each split. To define these hyperparameters (i.e., number of trees, number of variable at each split) we systematically varied the number of trees (100–1000), and number of variables selected for decision making (3–5) and tested the model accuracy with respect to the out-of-bag sample (re-defined during each iteration step). To ensure model-stability, the random forest regression was re-iterated 10 times with varying seeds. Please see SI 1.3.4 for details on parameters and details on statistical procedures. The first model investigating differential timing effects of ACE severity on brain volume contained the following predictor variables (i.e*. timing model*): timing – specific predictors, i.e. ACE severity at each year of life during the recollected lifespan (*time-specific ACE severity at* 3–17 years of age), as well as global predictors, i.e. *global ACE severity* (averaged severity across age 3 up to 17), and *group* (presence of a PTSD diagnosis or not). In a second model, the influence of the ACE type in interaction with the timing on brain volume was tested (i.e. *type and timing model*), i.e. the influence on abuse or neglect during differential time periods on brain volume. This second model contained the following predictor variables: type and timing specific predictors, i.e. *time-specific abuse severity*, as well as *time-specific neglect severity* during the recollected lifespan (age 3–17), as well as the global predictor variables, i.e. *global neglect* severity and *global abuse severity* (averaged neglect and abuse severity across age 3 up to 17), and *group*.

## Results

### Global ACE severity and regional brain volume

In general, regional brain volume was estimated and adjusted with respect to the current age of the participant, respectively. A negative association between *global ACE severity* across childhood and adolescence and bilateral amygdala volume was observed at a trend level (left: r = −0.23, *p* = 0.061, right: r = −0.216, *p* = 0.076, averaged amygdala volume: r = −0.23, *p* = 0.059). No significant associations were found regarding hippocampus or ACC volume (*p*-values > 0.117) (explorative analyses on effects of ACE and PTSD on brain volume can be found in SI 2.3).

### Severity of ACE type and regional brain volume

**Figure 1:**
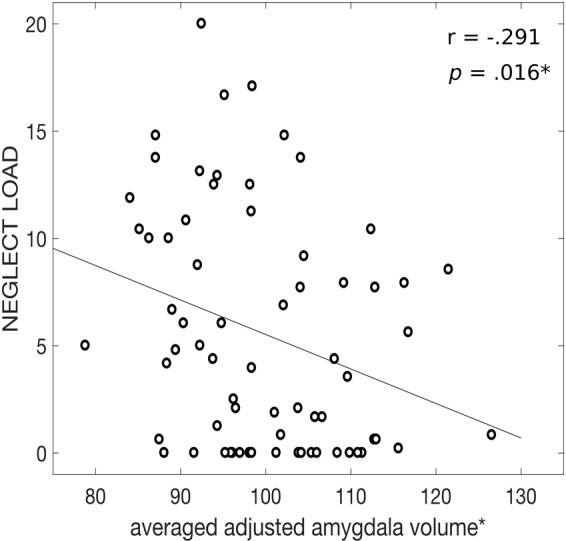
Linear regression graphs illustrate the relationship between the global neglect severity during childhood and adolescence and the averaged adjusted amygdala volume. *Brain volume adjusted for current age.

A negative association between *global neglect severity* across childhood and adolescence and the bilateral amygdala, as well as a trend regarding bilateral hippocampal volume was observed (amygdala: left: r = −0.26, *p* = 0.036, right: r = −0.31, *p* = 0.011, averaged amygdala volume: r = −0.29, *p* = 0.016; Fig. 1; hippocampus: left: r = −0.22, *p* = 0.067, right: r = −0.22, *p* = 0.073, averaged hippocampus volume: r = −0.23, *p* = 0.064). No significant associations were found regarding ACC volume (*p*-values > 0.449). No significant associations were observed between *global abuse severity* across childhood and adolescence, and brain volume (*p*-values > 0.622).

### ACE timing and regional brain volume

#### Amygdala

**Figure 2:**
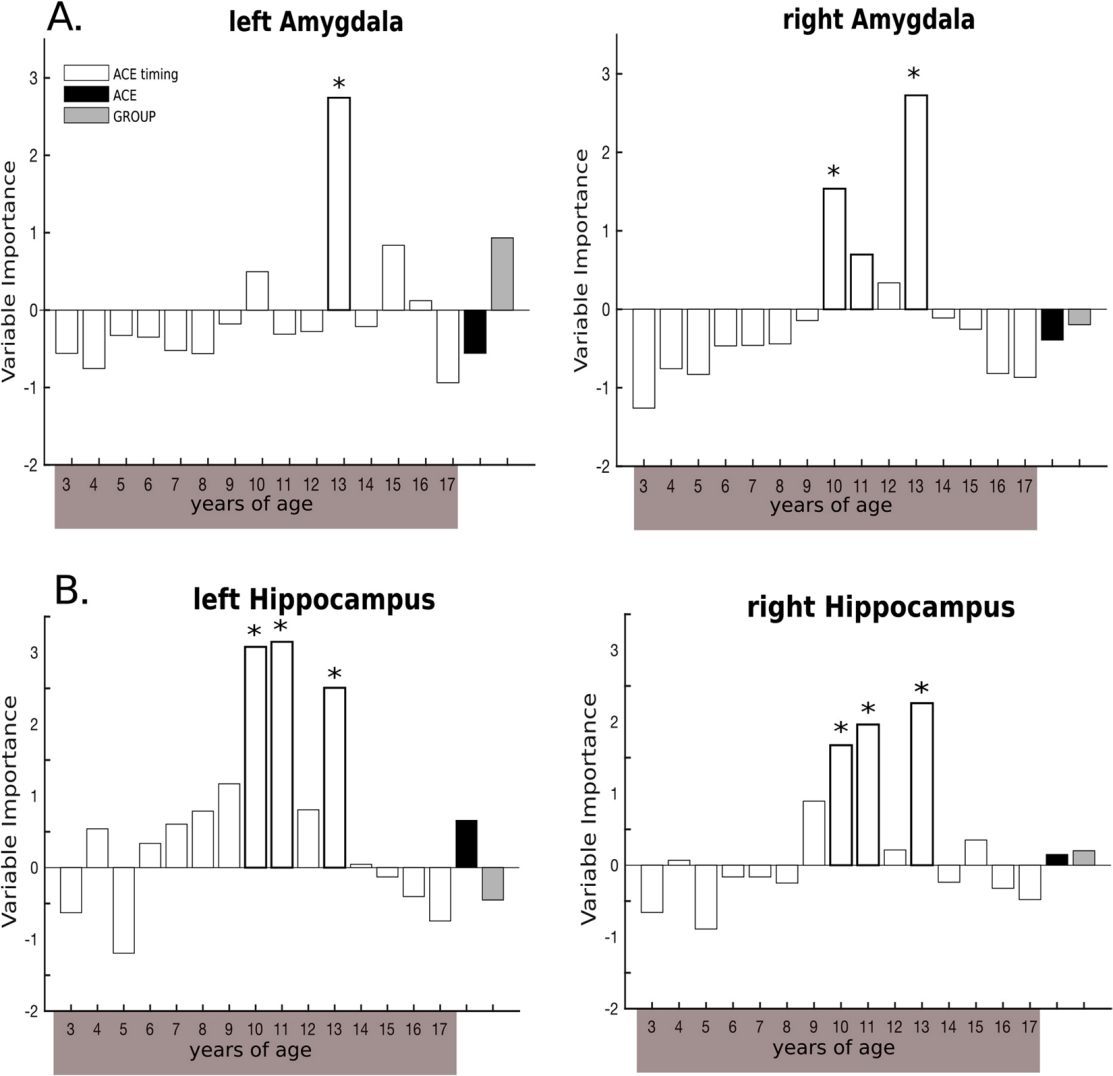
Results of random forest regression with conditional interference trees indicating the importance of time-specific ACE severity from 3 up to 17 years of age on bilateral amygdala (**A**), and hippocampal volume (**B**). Permutation test: *p < 0.05; ACE = adverse childhood experience.

Analyses of timing effects revealed that *time-specific ACE severity* at 13 years of age was an important predictor of both, left, and right adjusted amygdala volumes, while *time-specific ACE severity* at age 10 was also important in predicting right amygdala volume. Global predictors (i.e. *global ACE severity* and *group*) were not detected as important predictors (Fig. 2A, for *p*-values of VI scores and trends see Table S6). The relationship between the identified age 13 and bilateral amygdala volume, as well as age 10 and right amygdala volume was best described by a linear as compared to a quadratic model: Higher ACE at the identified ages was associated with lower bilateral amygdala volume (for statistics see Table S8).

#### Hippocampus

*Time-specific ACE severity* at 10, 11, and 13 years of age were important predictors for both, left, and right adjusted hippocampal volumes. Global predictors were not detected as important predictors (Fig. 2B, for *p*-values of VI scores and trends see Table S6). Illustrative analyses of the type of the relationship between identified ages and hippocampal volume revealed as a trend that the relationship between left hippocampal volume and *time-specific ACE severity* at 10 was best described by a linear model, while no significant linear or quadratic association was observed regarding *time-specific ACE severity* at 11 and 13 years of age. With respect to right hippocampal volume and identified ages 10, 11 and 13, a linear model was found to describe the relationship best, suggesting that higher ACE during the latter lifespan is associated with lower bilateral hippocampal volume (Table S8) (explorative analyses on ACC volume can be found in SI 2.4, SI 2.5).

### Severity of ACE type & timing and regional brain volume

#### Amygdala

**Figure 3:**
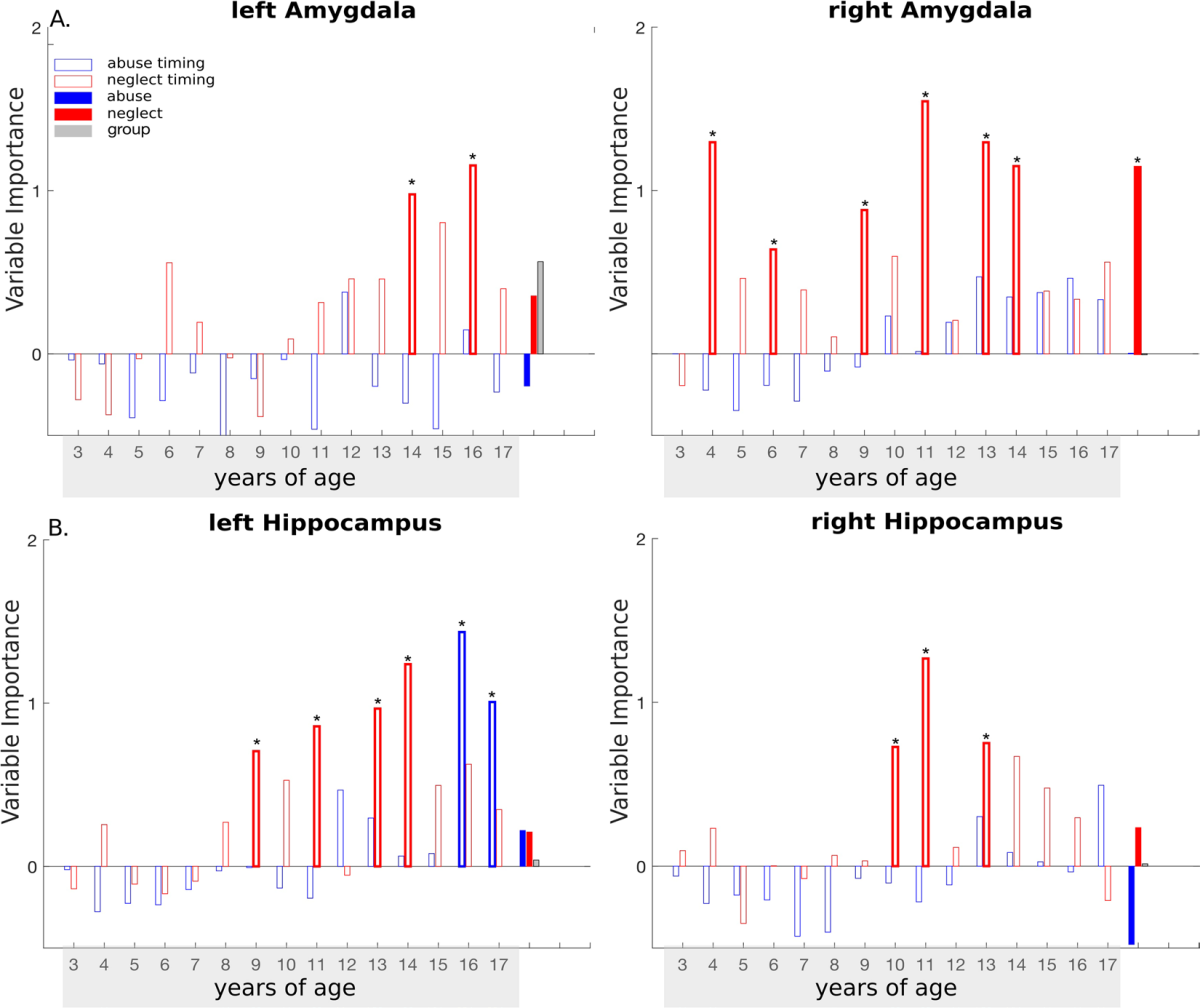
Results of random forest regression with conditional interference trees indicating the importance of time-specific neglect and abuse severity from 3 up to 17 years of age on bilateral amygdala (**A**), and hippocampal volume (**B**). Permutation test: *p < 0.05; ^†^<0.1.

Analyses of timing effects revealed that *time-specific neglect severity* at 14, and 16 years of age were important predictors of left amygdala volume. *Time-specific neglect severity* at 4, 6, 9, 11, 13, and 14 years of age predicted right adjusted amygdala volume (Fig. 3A, for *p*-values of VI scores and trends see Table S7). With respect to global predictors, *global neglect severity* was found to be an important predictor of right amygdala volume (Table S7). Post hoc analyses revealed that the relationship between the bilateral amygdala volume and the identified ages were best described by a linear model, suggesting that higher *time-specific neglect severity* was associated with lower bilateral amygdala volume (Table S8).

#### Hippocampus

*Time-specific abuse* at 16 and 17 years of age as well as *time-specific neglect severity* at 9, 11, 13 and 14 years of age were important predictors of left hippocampal volume. *Time-specific neglect severity* at 10, 11, and 13 years of age were important predictors of right adjusted hippocampal volume. Global predictors were not detected as important predictors (Fig. 3B, for *p*-values of VI scores and trends see Table S7). Post hoc analyses revealed that the relationship between the bilateral hippocampus volume and the identified ages were best described by a linear model: While higher *time-specific neglect severity* was related to a lower bilateral hippocampal volume, higher *time-specific abuse severity* was related to a greater left hippocampal volume (Table S8) (explorative analyses on ACC volume can be found in SI 2.4, SI 2.5).

## Discussion

The present study investigated alterations in brain volume related to retrospectively reported ACE in an adult female traumatized sample with an emphasis on differential effects of severity of type and timing of ACE on brain volume. We found a significant association between *global ACE severity* and bilateral amygdala volume, while we did not find any association between *global ACE severity* and hippocampal or ACC volume. The present findings highlight that the application of the dimensions of passive and active maltreatment^10,44^ can be of importance when investigating effects of ACE on brain volume: The association between *global ACE severity* and bilateral amygdala volume was driven by the passive maltreatment severity: Higher *global neglect severity* was associated with smaller bilateral amygdala volume, and at trend level with smaller bilateral hippocampal volume across traumatized individuals, while no such associations were observed for *global abuse severity*.

Studies so far have revealed heterogeneous findings regarding the direction of the relationship between the severity of neglect and amygdala volume, with some hinting towards a negative relationship^48,49^, while others provided evidence for a positive^14,28,50^ or no association^16,51^. These heterogeneous findings have been discussed in the context of type of ACE in modulating the relationship, as well as the chronicity and time elapsed since traumatization: Increased amygdala volume was observed primarily in children and adolescent samples with early exposure to emotional and/or physical neglect (but see^16,51^:, while studies reporting reductions in amygdala volume were related to older participants, greater degrees of psychopathology, and exposure to multiple types of abuse during childhood^7^). Therefore, it has been hypothesized that early exposure to ACE may result in an initial increase in amygdala volume, particularly noticeable during childhood, and/or may also sensitize the amygdala to further stress. The latter may result in a substantial reduction in amygdala volume most noticeable in late adolescence or adulthood^7^. This argumentation is in line with the present investigation, as our sample included adults with experience of prolonged and severe maltreatment. With regard to the hippocampus, a number of studies found reduced hippocampal volume in adult samples^23^, while studies in children or adolescents exposed to neglect have not typically observed changes in hippocampus volume^16^. As observed in the amygdala, it is hypothesized that there may be a silent period between exposure to maltreatment and discernible neurobiological differences, with observable cross sectional differences becoming fully discernible in later life^21^. This is further supported by animal studies, showing that early ACE can lead to an increase in certain brain regions immediately following the exposure; while these initial increases can be followed by shrinkage^21^. In light of heterogeneous findings, capturing maltreatment as an overall measurement, i.e. one score across early life, might be not detailed enough to capture more complex relationships.

Therefore, the present investigation highlights the importance of *time-specific ACE severity* having an impact on brain development. Timing analyses provided evidence for differential timing effects, during which *time-specific ACE severity* has an higher impact on brain volume: An effect of timing was observed covering preadolescence (10–11 years of age) and early adolescence (13 years of age), for both bilateral amygdala and hippocampal volume. This fine-grained analysis of differential timing effects matches those observed in previous studies, which also detected timing effects of ACE at the end of childhood and early adolescence^18,28^. Importantly, and besides brain development, similar time windows have been observed for ACE in fostering dissociative symptoms and PTSD symptoms, strengthening the idea that this time of development may be extremely vulnerable to the impact of ACE^52^ (for depressive symptomatology see also^53^). The importance of pre- and early adolescence as a time for the higher impact of ACE is further stressed by studies focusing on brain connectivity patterns across childhood and adolescence: A marked change in amygdala-cortical coupling has been found during the transition from childhood to adolescence, i.e. preadolescence (9–12 years of age), with no connectivity observed in childhood, while a negative coupling has been found at around 11 years of age^54^.

Narrowing down the influence of timing and additionally focusing on the severity of type in particular, provided a more detailed picture regarding the contribution of neglect in relation to abuse severity across the early life span. We decided to make a first distinction between neglect i.e. deprivation, and abuse i.e. threat, as it is a prominent model of adversity and thus provides a promising first step in delineating particular effects^44,55^. Distinct consequences have been assumed: Neglect comprises the absence of adaptive inputs, whereas abuse represents harmful experiences compromising the physical integrity^55^. Putting these in the context of timing effects, one may hypothesize that both forms may influence neuroanatomical measures differently. Indeed, we did observe type-related effects during different time periods, which were further distinguished in terms of brain structure: Regarding neglect severity and amygdala volume, vulnerable time windows were detected during preadolescence (10 and 12 years of age) and during adolescence (13 and 14 years of age) for right amygdala volume as well as during later adolescence (age 14 and 16 years of age) for the left amygdala volume. Thus both, pre-, and adolescence and a peak during late adolescence appear to be vulnerable to the severity of neglect. Likewise, we found a differential timing effect of pre-and early adolescence (9–13 years of age), affecting bilateral hippocampal volume in terms of neglect. Contrary to the findings of several studies and meta-analyses^8,56^, we did not find reduced hippocampal volume in subjects after childhood abuse. Our results even show a positive correlation between abuse and hippocampal volume. On the other hand, we found a negative correlation between neglect and hippocampal volume. Possibly due to the overall stronger influence of neglect compared to abuse, we also found an overall negative correlation between ACE severity and hippocampal volume. In earlier studies, the missing distinction between abuse and neglect might have blurred these differential findings.

### Limitations

There are several limitations that should be acknowledged. First, the present analyses of type and timing was based on retrospective reports which are prone to several potential recall biases. Due to the cross-sectional design of our study, we were not able to assess prospective data and objective confirmation of maltreatment (e.g., emergency room records, court filings). The suitability of retrospective measures of childhood maltreatment has recently been investigated and discussed in a meta-analysis by Baldwin and colleagues^57^ (for further discussion, please see^58^). The meta-analysis revealed poor agreement between prospective and retrospective measures of childhood maltreatment. Although the authors highlighted that prospective data are generally more advantageous from a scientific perspective to address causality^58^, they also highlighted that these results cannot directly be interpreted to indicate poor validity of retrospective measures. Prospective measures are often characterized by a lower sensitivity, as official records often capture only the most severe cases of maltreatment. Moreover, the meta-analysis revealed that the agreement between prospective and retrospective reports is higher in investigations a) applying interviews instead of questionnaires to elicit ACE, b) studying small sample sizes, and c) providing a precise definition of childhood maltreatment. The current investigation did indeed assess childhood maltreatment via the extensive MACE interview, examining 10 different and well defined types of ACE during each year of childhood and adolescence from 3 up to 17 years of age. The interview has been conducted by well-trained and specialized clinical psychologists. The psychometric evaluation of the German version of the MACE provides good support for a valid and detailed assessment of ACE (please see^42,43^). Moreover, the good test–retest reliability of the MACE provides support that adults are very consistent in their recall of the timing of maltreatment experiences, as such events are often a vital part of an individual’s personal narrative^42,43^. Furthermore, the relatively small sample size in our study allowed an intensive support for participants, resulting in a greater engagement of participants and a detailed retrospective assessment of ACE by diagnosticians. Critically, rapid brain development has been reported during 0–3 years of age, which might leads to pronounced vulnerability towards the impact of ACE during this time^59^. However, verbal autobiographical memories are more accessible from three years of age onward; therefore we decided to investigate the influence of ACE from 3 up to 17 years of age in the present investigation. Moreover, it is important to mention that children reared in maltreating circumstances are also likely to experience a number of ongoing additional stressors, such as poverty, dysfunctional parent-child interaction, which in addition might impact brain development^34^. In the same context, we further have not assessed protective factors, which possibly might have also an impact of neuroanatomical measures. Adding to this complexity, the impact of ACE on an individual’s neurobiology needs further consideration in the context of genetic and epigenetic processes. Although, a detailed consideration of gene-environment interaction is beyond the scope of our study, a particularly relevant area of research are studies of gene-environment interaction of the FKBP5 gene with ACE, which regulates cortisol-binding affinity and the nuclear translocation of the glucocorticoid receptor (for the interested reader see^60–63^:). Furthermore, we only included female participants. One has to keep in mind that animal as well as human work on ACE points towards differential effects of ACE in male and females^23,64^. Therefore our results are limited to females and cannot be generalized to male subjects. Finally, we have not assessed behavioral data, which prohibits to analyze potential important relationships between the present structural associations and ACE (e.g., with the hippocampus) and behavioral measures such as memory performance.

Going forward, we urgently need longitudinal and prospective designs including male and female individuals, to better understand the impact of ACE across the entire lifespan on neuroanatomical and behavioral measures. More precisely, future longitudinal studies are urgently needed, focusing on the identification of potential important variables, such as environmental protective factors, objective measurements of maltreatment, gene-environment interaction that may modulate the relationship of functional and structural brain characteristics and ACE leading to potential cognitive, emotional and behavioral alterations. Addressing these questions is the aim of our ongoing graduate program ‘Impact of Adverse Childhood Experiences on Psychosocial and Somatic Conditions across the Lifespan’ (GRK2350).

## Conclusion

The present study explored the relationship between stress-sensitive brain structures and the effect of severity of type and timing of reported ACE in an adult female traumatized sample. Timing analyses provided evidence for a timing effect covering pre- and early adolescence in influencing amygdala and hippocampal volume. Extending the timing analysis and focusing on the predictive power of ACE type in relation to timing of ACE, we found differential timing effects of abuse and neglect for amygdala and hippocampal volume, respectively. The present results strengthen the idea of a type- and time-sensitive model of ACE in terms of brain volume. This is an important step in gaining a better understanding how early life adversity affects neurodevelopment in terms of providing insight into differential time windows during which ACE has an highly deleterious effect on neuroanatomical measures.

## Supplementary information

Supplementary Info.

## Data Availability

The datasets generated during and/or analyzed during the current study will be available at https://osf.io/kt7qr/?view_only=16181bf2e6db41cf906f46e192bfc073.
