# Supplementary material for: Influence of Severity of Type and Timing of Retrospectively Reported Childhood Maltreatment on Female Amygdala and Hippocampal Volume
Source: Sci Rep. 2020 Feb 5;10:1903. doi: 10.1038/s41598-020-57490-0 (PMC7002661; doi:10.1038/s41598-020-57490-0)
Supplement: Supplementary file 1 — Supplementary Info. [file 41598_2020_57490_MOESM1_ESM.pdf]

SUPPLEMENTAL INFORMATION:

INFLUENCE OF SEVERITY OF TYPE AND TIMING OF ADVERSE CHILDHOOD EXPERIENCES ON HUMAN AMYGDALA AND HIPPOCAMPAL VOLUME

Julia I. Herzog[a, *, §] MSc, Janine Thome[b,d,e,f, $] PhD, Traute Demirakca[c] PhD, Georgia Koppe[d,e] PhD, Gabriele Ende[c] PhD, Stefanie Lis[b] PhD, Sophie Rausch[b] MSc, Kathlen Priebe[g] MSc, Meike Müller-Engelmann[h] PhD, Regina Steil[h] PhD, Martin Bohus[b] MD and Christian Schmahl[a,f] MD

[a] Department of Psychosomatic Medicine and Psychotherapy, Central Institute of Mental Health Mannheim, Medical Faculty Mannheim, Heidelberg University, J5, 68159 Mannheim, Germany; [b] Institute for Psychiatric and Psychosomatic Psychotherapy, Central Institute of Mental Health Mannheim, Medical Faculty Mannheim, Heidelberg University, J5, 68159 Mannheim, Germany; [c] Department of Neuroimaging, Central Institute of Mental Health Mannheim, Central Institute of Mental Health Mannheim, Medical Faculty Mannheim, Heidelberg University, J5, 68159 Mannheim, Germany; [d] Department of Theoretical Neuroscience, Central Institute of Mental Health Mannheim, Medical Faculty Mannheim, Heidelberg University, J5, 68159 Mannheim, Germany; [e] Department of Psychiatry and Psychotherapy, Central Institute of Mental Health Mannheim, Medical Faculty Mannheim, Heidelberg University, J5, 68159 Mannheim, Germany; [f] Department of Psychiatry, University of Western Ontario, 339 Windermere Rd., London, N6A 5A5 ON, Canada; [g] Department of Psychology, Faculty of Life Sciences, Humboldt-University of Berlin, Unter den Linden 6, 10999 Berlin, Germany; [h] Department of Clinical Psychology and Intervention, Institute of Psychology, Goethe-University Frankfurt, Varrentrappstr. 40-42, 60486 Frankfurt am Main, Germany

*Corresponding Author: J. I. Herzog, Department of Psychosomatic Medicine and Psychotherapy, Central Institute of Mental Health, J 5, 68159 Mannheim, Germany. Tel: +49 0621 1703 4402. Email: Julia.herzog@zi-mannheim.de

§ Both authors contributed equally to this work

This file includes:

Supplementary text

Figs. S1 to S4

Tables S1 to S9

1. Supplemental Methods

1.1 Participants

1.1.1 Diagnostic and consenting procedures

Clinical diagnoses were assessed by trained diagnosticians using the Structure Clinical Interview for DSM-IV Axis I Disorders SCID-I; [1], the Clinician Administered PTSD Scale CAPS; [2], and the BPD section of the International Personality Disorder Examination IPDE; [3]. Self-report measures included retrospective questionnaires on childhood trauma (Childhood Trauma Questionnaire; CTQ; [4], PTSD symptomatology (Davidson Trauma Scale; DTS; [5], and severity of depressive mood (Beck Depression Inventory; BDI-II; [6]. Details on demographic data and clinical characteristics of the sample are reported in Table S1. The study was approved by the Ethical Board II of Heidelberg University, Germany. It was conducted according to the Declaration of Helsinki at the Central Institute of Mental Health in Mannheim. Written informed consent was obtained from the participants after the procedure had been fully explained. All participants received monetary remuneration for participation in the study.

1.1.2 Inclusion and exclusion criteria

Participants with posttraumatic stress disorder (PTSD) were recruited from a larger randomized controlled trial evaluating dialectical behavioral therapy for PTSD (DRKS00010827). Trauma exposed healthy control participants (TC) were recruited via advertisements in local newspapers, flyers and internet. Exclusion criteria for all participants were metal implants, pregnancy, left-handedness, and claustrophobia. Exclusion criteria for PTSD participants covered current and lifetime schizophrenia or bipolar-I disorder, mental retardation, severe psychopathology, traumatic brain injuries or somatic illness that needs to be treated immediately in another setting (e.g., BMI<16), medical conditions making exposure-based treatment impossible, a suicide attempt within the last two months, and substance dependency with no abstinence within two months prior to the study. Exclusion criteria for the TC sample were any current or previous mental disorder, any psychotherapeutic experience or any intake of psychotropic medication for more detailed descriptions of the TC sample see: [7].

1.2 Measures

1.2.1 Maltreatment exposure

The time course and severity of exposure to traumatic events was assessed using an adapted version of the Maltreatment and Abuse Chronology of Exposure interview (MACE; [8,9]. The inventory evaluates ten types of adverse childhood experiences (emotional neglect, physical neglect, parental physical abuse, siblings physical abuse, parental emotional abuse, siblings emotional abuse, sexual abuse, peer abuse, witnessing interparental violence and witnessing violence to siblings) during each year of childhood 3 up to age 17. Scores can be calculated for each ACE type, as well as a total score based on the sum score of all categories. Moreover, the duration, as well as the amount of ACE types experienced during childhood and adolescents can be calculated. With respect to the MACE severity score, test-retest reliability over a time period of 6 month has been found to be very reliable in an US population (Severity: r=.91 [95% CI 0.86-0.94]; p values < .001) [8]. Convergent validity scores were found to be good as the MACE severity score correlated 0.74 (95%, CI =0.69– 0.78, $p < 10^{-16}$) with the Childhood Trauma Questionnaire (CTQ) scores and 0.71 (95%, CI = 0.68– 0.73, p < .001) an US population [8]. The German version has also been tested and the convergent validity scores were found to be comparable (CTQ, r = 0.75, p < .001) [9]. Within the present investigation, ACE was quantified by a) an averaged MACE severity score indicating ACE across childhood and adolescence, (i.e. *global ACE severity*), and for each year of life, respectively (i.e. *time-specific ACE severity*) [8]. The scores range from 0 to 100. To address b) the conceptual framework of active and passive maltreatment [10,11], we created two dimensions: Active maltreatment is represented by collapsing the subscales physical and sexual abuse (= *abuse*), while passive maltreatment is represented by collapsing the subscales physical and emotional neglect (= *neglect*). The scores have been averaged across childhood and adolescence, i.e. *global abuse severity*, and *global neglect severity*, as well as for each year of life, respectively i.e. *time-specific abuse severity*, and *time-specific neglect severity*. The neglect and abuse score ranges from 0 to 20.

1.2.2 Magnetic resonance imaging

Data was collected using a Siemens 3 Tesla TRIO-Scanner (Siemens Medical Solutions, Erlangen, Germany) with a 12-channel head coil. Using three-dimensional magnetisation-prepared rapid-acquisition gradient echo (MPRAGE; T1-weighted contrast, TE: 30 ms; TR: 2000 ms; FA = 80°; FOV: 192 x 192 mm; number of slices 176, voxel size 1x1x1 mm³), a high-resolution anatomical scan was acquired for each participant. Head movement artefacts and scanning noise were restricted using head cushions and headphones.

1.2.3 Image processing

Preprocessing of the anatomical T1 images was conducted in Statistical Parametric Mapping (SPM12; http://www.fil.ion.ucl.ac.uk/spm/), and images were segmented into grey matter volume (GM), white matter

volume (WM), and cerebrospinal fluid (CSF). Whole brain volume of different compartments was determined by integrating all voxels of GM, WM volume and CSF images. Subsequently, the individual images were normalized to an IXI550 template (McConnell Brain Imaging Centre). The voxel values were modulated with the Jacobian determinant to preserve the amount of change during normalization. Additionally, regions of interest (ROI), i.e. the bilateral amygdala, hippocampus, and anterior cingulate cortex were defined using the WFU Pickatlas (http://fmri.wfubmc.edu/software/pickatlas). The volume of each ROI was estimated, via the integration of all voxel values within the ROI of the GM image. This was conducted for each subject and the estimated size of each ROI was related to the individuals total intracranial volume (GM+WM+CSF = TIV). Regional volumes corrected for TIV, as well as GM, and WM volume were extracted and exported into SPSS (version 23; SPSS Inc., USA), R (version 3.3.3, and Matlab (Matlab R2016b, Simulink) for statistical analyses. Brain volume estimates were further corrected for current age, i.e. the current age was regressed out and residuals were z-transformed and taken for further analyses.

1.3 Supplemental Statistical Analyses

1.3.1 Across Group Analyses

To test, whether the amount of ACE severity differed across the recollected life-span, i.e. 3 up to 17 years of age, a repeated measurement ANOVA (rmANOVA) was applied with the within-subject factor ‘age’ (3–17). To investigate, whether the amount of traumatization in relation to the type differed across the recollected life-span, a rmANOVA with the within-subject factor ‘age’ (3-17) and ‘type’ (abuse, neglect) was applied. To assess the relationship between global ACE severity, i.e. averaged ACE severity across the recollected life-span, as well as with respect to global ACE type, i.e. *global neglect severity* and *global abuse severity*, and brain volume (amygdala, hippocampus, ACC volume corrected for TIV and age), Pearson correlations were conducted.

1.3.2 Between Group Analyses

To exploratory test, whether the presence of a PTSD diagnosis has an impact on the observed results, PTSD participants were contrasted to TC participants. Sample characteristics, i.e. sociodemographic variables (age, years of education), clinical characteristics (CTQ, DTS, MACE), were compared with t - statistics (Table S5). To test whether the groups differed with respect to the amount of ACE severity across the reported life-span, i.e. 3 up to 17 years of age a rmANOVA was applied with the between-subject factor ‘group’ and the within-subject factor ‘age’ (age 3 up to 17). To investigate, whether the amount of traumatization in relation to the type differed across the life-span between the groups, a rmANOVA with the between-subject factor ‘group’, and the within-subject factor ‘age’ (3-17) and ‘type’ (abuse, neglect) was applied. Neuroimaging measures with respect to each TIV-adjusted regional ROI were analysed in separate rmANOVA with ‘group’ as between-subjects factor, and ‘hemisphere’ as within-subject factor and the covariate ‘age’.

1.3.3 General information

For further description of statistical effects in the ANOVA designs, post-hoc comparisons were calculated - if appropriate - by pairwise comparisons (Bonferroni-adjusted for multiple testing). Statistical significance was set to $p < .05$. All analyses were performed using SPSS (version 23; SPSS Inc., USA).

1.3.4 Sensitive period analysis

To test the presence of sensitive periods in which exposure to ACE might be related to alterations in ROI brain volume, we applied random forest regression with conditional interference trees (‘cforest’ in R package ‘party’ [12,13]. This is a machine learning approach, in which an ensemble of unpruned regression trees (forest) is generated. This method is advantageous compared to conventional linear modelling to identify important predictors, as conditioned forest regression considers multicollinearity between predictor variables, does not require specific distribution assumptions or a definition of the relationship between the predictor and response, and can handle a large number of predictors modelling the outcome [13-15]. With respect to the concept how the forest is created, tree building particularly is based on the principle of recursive partitioning, meaning that the feature space (= space spanned by all predictor variables) is recursively partitioned in such that observations with similar response values are grouped [12,13]. Thus, smaller groups are generated, which are more homogenous with respect to the outcome. As a single decision tree provides a good fit to the data but is typically a weak predictor in regard to its generalizability, prediction in random forest regression is therefore improved by aggregating trees. Importantly, each tree in the forest is unique, as each tree is generated based on a subset of the entire dataset (bagging), while also the number of predictor variables available at each decision point is restricted. Predictive performance of the model is estimated on the sample that is left out (out-of-bag sample) and thus random forest regression provide an internal estimate, which has found to be highly correlated with either cross-validation or test set estimates [14,15]. Importance of a given predictor is identified by the variable importance score (VI) [12,13]: The score refers to the decrease in model accuracy following the permutation of a given predictor variable. Thereby, if the permutation of a predictor variable causes model accuracy to decrease, it is considered “important”, i.e. it has a higher VI score, while if permuting has no or little impact on model accuracy it is also

not considered as "important". Each random forest model consisted of 500 trees with 4 variables randomly selected for decisions making at each node [16]. To identify, whether the magnitude of VI could have occurred by chance, we applied permutation tests in which the outcome measure (ROI volume) was permuted 1000 times and VI scores for each predictor were assessed [17]. *P*-values were determined in terms of the empirical distribution (by the fraction of permutation-based VI scores greater than the not permuted score) [17,18]. It has to be noted that random forest regression does not provide information on the nature of the relationship, as it is a machine learning algorithm aiming at the detection of relevant predictors, with no a priori assumption of the type of the relationship and thus also considering complex relationships (linear, nonlinear, interaction between predictor variables) . To illustrate the relationship, we therefore examined whether the identified predictor variables and brain volumes might be significantly linearly or quadratically related, while it has to be kept in mind that the relationship may be also more complex. To test the latter, we investigated whether the relationship between the identified ages and ROI volume could better be described by a linear or quadratic model. We set up two general linear models (GLM), one containing a single linear predictor variable, and the second containing an additional quadratic term. To test whether the quadratic term significantly added to the understanding of the relationship between brain volume and identified ages, we tested whether the amount of additional variance explained by the quadratic term (second model) was significant via the F-distribution [19].

2. Supplemental Results

2.1 Maltreatment exposure history

Traumatized subjects reported a history of prolonged traumatization during childhood and adolescence (number of years: mean =12.81, SD =3.42), while they were exposed to a variety of ACE types (mean =6.01, SD =2.34) (Table S4) (for differences between PTSD and TC participants please see SI 2.2 and Table S5). A detailed characterization of the *ACE severity* revealed that the amount of traumatization differed across years of age ($F(14,938) = 32.19$, $p < .001$, Figure S1A): ACE severity at the beginning, i.e. age 3-6, as well as at the end of the recollected time span, i.e. age 15-17, was lower than during most of the remaining ages (*p*-values < .045). A significant interaction between severity of type and timing (type x age: $F(14,938) = 7.84$, $p < .001$, Figure S1B) revealed that participants reported higher neglect compared to abuse severity at age 3 ($p < .01$) and between 12 and 17 years of age (*p*'s < .035). Abuse severity at the beginning, i.e. age 3-5 of the recollected life span and at the end of the recollected life span, i.e. age 15-17, was lower than for most of the remaining years of age (*p*-values < .022). With respect to the neglect severity, the reported neglect at the beginning, i.e. age 3-5, as well as the end of the recollected life span, i.e. age 16, and 17 was lower than for most of the remaining years of age (*p*-values < .039). For detailed comparisons, respectively, please see Table S1 (global ACE severity), Table S2 (global abuse severity) and Table S3 (global neglect severity).

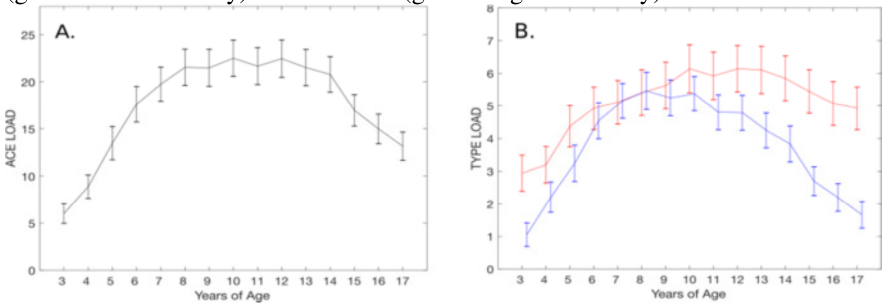

Figure S1 Chronology of ACE regarding ACE severity (A.), and severity of ACE type, i.e. abuse (blue), and neglect (red) (B).

2.2 Maltreatment exposure history and clinical characteristics: Group Comparison

For differences in socio-demographic and clinical characteristics please see Table S5. In general, both groups reported exposure to various trauma types (PTSD: AM = 6.83, SD = 2.24; TC: AM = 4.69, SD = 1.85), as well as exposure to maltreatment for a long time period (PTSD: AM = 13.79, SD = 2.76; TC: AM = 11.23, SD = 3.83). Contrasting both groups revealed that PTSD participants reported more trauma types, as well as a longer period of traumatization compared to trauma controls (Table S5). Contrasting both groups with respect to the *global ACE severity* across the recollected lifespan revealed that PTSD compared to TC individuals reported more ACE, while this effect was influenced by years of age (group: $F(1,66) = 28.03$, $p < .001$; group x age: $F(14,924) = 3.00$, $p < .001$). Taking the *severity of type of ACE* (*global abuse severity* vs *global neglect severity*) into account, while contrasting both groups revealed that groups differed with respect to *global ACE severity* in general (group: $F(1,66) = 24.78$, $p < .001$), while this was further a trend towards the influence of the type (group x type: $F(1,66) = 3.67$, $p = .060$): PTSD participants reported both, more abuse as well as neglect compared to

TC participants ($p < .001$). While PTSD participants reported more neglect compared to abuse ($p = .002$), TCs did not differ regarding the recollected amount of abuse compared to neglect ($p = .963$).

Table S1. Bonferroni adjusted post-hoc comparison of the time course of global ACE severity

Age	3		4		5		6		7		8		9		10		11		12		13		14		15		16		17	
**3**			0.00	4>	0.00	5>	0.00	6>	0.00	7>	0.00	8>	0.00	9>	0.00	10>	0.00	11>	0.00	12>	0.00	13>	0.00	14>	0.00	15>	0.00	16>	0.00	17>
**4**	0.00	3<			0.09		0.00	6>	0.00	7>	0.00	8>	0.00	9>	0.00	10>	0.00	11>	0.00	12>	0.00	13>	0.00	14>	0.00	15>	0.01	16>	0.25	
**5**	0.00	3<	0.09				0.00	6>	0.00	7>	0.00	8>	0.00	9>	0.00	10>	0.00	11>	0.00	12>	0.00	13>	0.00	14>	1.00		1.00		1.00	
**6**	0.00	3<	0.00	4<	0.00	5<			0.03	7>	0.00	8>	0.04	9>	0.00	10>	0.06		0.02	12>	0.36		1.00		1.00		1.00		0.43	
**7**	0.00	3<	0.00	4<	0.00	5<	0.03	6<			0.46		1.00		0.63		1.00		1.00		1.00		1.00		1.00		0.25		0.00	17<
**8**	0.00	3<	0.00	4<	0.00	5<	0.00	6<	0.46				1.00		1.00		1.00		1.00		1.00		1.00		0.55		0.02	16<	0.00	17<
**9**	0.00	3<	0.00	4<	0.00	5<	0.04	6<	1.00		1.00				1.00		1.00		1.00		1.00		1.00		0.26		0.00	16<	0.00	17<
**10**	0.00	3<	0.00	4<	0.00	5<	0.00	6<	0.63		1.00		1.00				1.00		1.00		1.00		1.00		0.01	15<	0.00	16<	0.00	17<
**11**	0.00	3<	0.00	4<	0.00	5<	0.06		1.00		1.00		1.00		1.00				1.00		1.00		1.00		0.07		0.00	16<	0.00	17<
**12**	0.00	3<	0.00	4<	0.00	5<	0.02	6<	1.00		1.00		1.00		1.00		1.00				1.00		1.00		0.00	15<	0.00	16<	0.00	17<
**13**	0.00	3<	0.00	4<	0.00	5<	0.36		1.00		1.00		1.00		1.00		1.00		1.00				1.00		0.02	15<	0.00	16<	0.00	17<
**14**	0.00	3<	0.00	4<	0.00	5<	1.00		1.00		1.00		1.00		1.00		1.00		1.00		1.00				0.04	15<	0.00	16<	0.00	17<
**15**	0.00	3<	0.00	4<	1.00		1.00		1.00		0.55		0.26		0.01	10>	0.07		0.00	12>	0.02	13>	0.04	14>			0.01	16<	0.00	17<
**16**	0.00	3<	0.01	4<	1.00		1.00		0.25		0.02	8>	0.00	9>	0.00	10>	0.00	11>	0.00	12>	0.00	13>	0.00	14>	0.01	15>			0.00	17<
**17**	0.00	3<	0.25		1.00		0.43		0.00	7>	0.00	8>	0.00	9>	0.00	10>	0.00	11>	0.00	12>	0.00	13>	0.00	14>	0.00	15>	0.00	16>		

Post-hoc t tests were performed at a significance level of p< .05 Bonferroni-corrected

Table S2. Bonferroni adjusted post-hoc comparison of the time course of global abuse severity

Age	3		4		5		6		7		8		9		10		11		12		13		14		15		16		17	
**3**			0.03	4>	0.00	5>	0.00	6>	0.00	7>	0.00	8>	0.00	9>	0.00	10>	0.00	11>	0.00	12>	0.00	13>	0.00	14>	0.07		0.92		1.00	
**4**	0.03	3<			0.88		0.00	6>	0.00	7>	0.00	8>	0.00	9>	0.00	10>	0.01	11>	0.01	12>	0.19		1.00		1.00		1.00		1.00	
**5**	0.00	3<	0.88				0.00	6>	0.00	7>	0.00	8>	0.00	9>	0.00	10>	0.41		0.58		1.00		1.00		1.00		1.00		0.60	
**6**	0.00	3<	0.00	4<	0.00	5<			0.31		0.40		1.00		1.00		1.00		1.00		1.00		1.00		0.17		0.02	16<	0.00	17<
**7**	0.00	3<	0.00	4<	0.00	5<	0.31				1.00		1.00		1.00		1.00		1.00		1.00		1.00		0.00	15<	0.00	16<	0.00	17<
**8**	0.00	3<	0.00	4<	0.00	5<	0.40		1.00				1.00		1.00		1.00		1.00		1.00		0.22		0.00	15<	0.00	16<	0.00	17<
**9**	0.00	3<	0.00	4<	0.00	5<	1.00		1.00		1.00				1.00		1.00		1.00		1.00		0.25		0.00	15<	0.00	16<	0.00	17<
**10**	0.00	3<	0.00	4<	0.00	5<	1.00		1.00		1.00		1.00				1.00		1.00		0.50		0.03	14<	0.00	15<	0.00	16<	0.00	17<
**11**	0.00	3<	0.01	4<	0.41		1.00		1.00		1.00		1.00		1.00				1.00		1.00		1.00		0.01	15<	0.00	16<	0.00	17<
**12**	0.00	3<	0.01	4<	0.58		1.00		1.00		1.00		1.00		1.00		1.00				1.00		0.33		0.01	15<	0.00	16<	0.00	17<
**13**	0.00	3<	0.19		1.00		1.00		1.00		1.00		1.00		0.50		1.00		1.00				1.00		0.26		0.01	16<	0.00	17<
**14**	0.00	3<	1.00		1.00		1.00		1.00		0.22		0.25		0.03	10>	1.00		0.33		1.00				0.94		0.22		0.00	17<
**15**	0.07		1.00		1.00		0.17		0.00	7>	0.00	8>	0.00	9>	0.00	10>	0.01	11>	0.01	12>	0.26		0.94				1.00		0.03	17<
**16**	0.92		1.00		1.00		0.02	6>	0.00	7>	0.00	8>	0.00	9>	0.00	10>	0.00	11>	0.00	12>	0.01	13>	0.22		1.00				0.17	
**17**	1.00		1.00		0.60		0.00	6>	0.00	7>	0.00	8>	0.00	9>	0.00	10>	0.00	11>	0.00	12>	0.00	13>	0.00	14>	0.03	15>	0.17			

Post-hoc t tests were performed at a significance level of p< .05 Bonferroni-corrected

Table S3. Bonferroni adjusted post-hoc comparison of the time course of global neglect severity

Age	3		4		5		6		7		8		9		10		11		12		13		14		15		16		17	
**3**			1.00		0.06		0.02	6>	0.01	7>	0.01	8>	0.00	9>	0.00	10>	0.00	11>	0.00	12>	0.00	13>	0.00	14>	0.00	15>	0.02	16>	0.06	
**4**	1.00				0.23		0.04	6>	0.02	7>	0.01	8>	0.01	9>	0.00	10>	0.00	11>	0.00	12>	0.00	13>	0.00	14>	0.01	15>	0.07		0.13	
**5**	0.06		0.23				1.00		1.00		0.54		0.33		0.01	10>	0.06		0.01	12>	0.05	13>	0.24		1.00		1.00		1.00	
**6**	0.02	3<	0.04	4<	1.00				1.00		1.00		1.00		0.02	10>	0.17		0.02	12>	0.32		1.00		1.00		1.00		1.00	
**7**	0.01	3<	0.02	4<	1.00		1.00				1.00		1.00		0.16		1.00		0.24		1.00		1.00		1.00		1.00		1.00	
**8**	0.01	3<	0.01	4<	0.54		1.00		1.00				1.00		0.41		1.00		1.00		1.00		1.00		1.00		1.00		1.00	
**9**	0.00	3<	0.01	4<	0.33		1.00		1.00		1.00				1.00		1.00		1.00		1.00		1.00		1.00		1.00		1.00	
**10**	0.00	3<	0.00	4<	0.01	5<	0.02	6<	0.16		0.41		1.00				1.00		1.00		1.00		1.00		1.00		0.78		0.31	
**11**	0.00	3<	0.00	4<	0.06		0.17		1.00		1.00		1.00		1.00				1.00		1.00		1.00		1.00		1.00		1.00	
**12**	0.00	3<	0.00	4<	0.01	5<	0.02	6<	0.24		1.00		1.00		1.00		1.00				1.00		1.00		1.00		0.13		0.04	17<
**13**	0.00	3<	0.00	4<	0.05		0.32		1.00		1.00		1.00		1.00		1.00		1.00				1.00		1.00		0.06		0.01	17<
**14**	0.00	3<	0.00	4<	0.24		1.00		1.00		1.00		1.00		1.00		1.00		1.00		1.00				1.00		0.14		0.04	17<
**15**	0.00	3<	0.01	4<	1.00		1.00		1.00		1.00		1.00		1.00		1.00		1.00		1.00		1.00				1.00		0.54	
**16**	0.02	3<	0.07		1.00		1.00		1.00		1.00		1.00		0.78		1.00		0.13		0.06		0.14		1.00				1.00	
**17**	0.06		0.13		1.00		1.00		1.00		1.00		1.00		0.31		1.00		0.04	12>	0.01	13>	0.04	14>	0.54		1.00			

Post-hoc t tests were performed at a significance level of $p < .05$ Bonferroni-corrected

Table S4. Demographic and clinical variables

	N=68	
Demographics		
age mean (SD)	35.06	(12.30)
years of education (SD)	10.88	(1.23)
Clinical Characteristics		
Childhood Trauma questionnaire (CTQ)		
Total (SD)	68.96	(22.29)
Abuse - total (SD)	25.15	(9.52)
Neglect - total (SD)	27.25	(9.89)
Emotional abuse (SD)	16.56	(5.76)
Physical abuse (SD)	11.18	(5.71)
Sexual abuse (SD)	13.97	(6.99)
Emotional neglect (SD)	16.73	(5.74)
Physical neglect (SD)	10.52	(4.84)
Davidson Trauma Scale (DTS)		
Total (SD)	52.85	(36.21)
Intensity (SD)	26.55	(19.11)
Frequency (SD)	26.06	(17.68)
Beck Depression Inventory 2 (BDI-II)		
Total (SD)	23.95	(18.54)
MACE		
Severity (SD)	17.53	(12.64)
Duration (SD)	12.81	(3.42)
Types (SD)	6.01	(2.33)
MACE Trauma Types		
Neglect (SD)	5.14	(5.11)
Abuse (SD)	3.77	(3.24)
Emotional Abuse Parents (SD)	3.58	(2.42)
Emotional Abuse Siblings (SD)	0.69	(1.38)
Physical Abuse Parents (SD)	2.84	(2.65)
Physical Abuse Siblings (SD)	0.44	(1.22)
Sexual Abuse (SD)	0.48	(0.57)
Emotional Neglect (SD)	3.50	(3.46)
Physical Neglect (SD)	1.64	(2.01)
Peer Abuse (SD)	1.43	(1.67)
Witnessing Abuse between Parents (SD)	1.09	(1.73)
Witnessing Abuse towards Siblings (SD)	1.83	(2.02)
Current Comorbidities N		
Posttraumatic Stress Disorder	42	
Affective Disorder	27	
Substance Dependency	0	
Substance Abuse	1	
Anxiety Disorder	30	
Obsessive Compulsive Disorder	7	
Somatization Disorder	3	
Eating Disorder	5	
Borderline Personality Disorder	20	
Psychotropic Medication N		
SSRI	11	
SNRI	10	

Tricyclica	5
Other Antidepressants	4
Neuroleptics	8
Anticonvulsants	3
Unmedicated	15

Legend: CTQ abuse - total (SD) = CTQ physical abuse + CTQ sexual abuse; CTQ neglect - total (SD) = CTQ emotional neglect + CTQ physical neglect. *MACE = Maltreatment and Abuse Chronology of Exposure scale, SD = standard deviation, severity = ACE severity averaged across age 3 up to 17 with respect of all ten types of ACE, duration = averaged years of traumatization reported across age 3 up to 17, types = average numbers of ACE types experienced between age 3 up to 17 (maximal value = 10)*

Table S5. Demographic and clinical variables in PTSD and trauma control subjects

	PTSD		TC		*Test-Statistics*				
	N=42		N=26		*T*	*df*	*p*		
Demographics									
Age mean (SD)	37.43	(11.80)	31.23	(12.36)	2.07	66	0.04	*	PTSD>TC
Years of education (SD)	10.62	(1.31)	11.31	(0.97)	-2.49	64	0.02	*	TC>PTSD
Clinical Characteristics									
Childhood Trauma questionnaire (CTQ)									
Total (SD)	78.49	(21.67)	53.57	(12.75)	5.97	65.89	<.001	*	PTSD>TC
Abuse - total (SD)	28.29	(10.12)	20.08	(5.64)	4.29	65.45	<.001	*	PTSD>TC
Neglect - total (SD)	31.63	(9.15)	20.18	(6.38)	5.59	66	<.001	*	PTSD>TC
Emotional abuse (SD)	18.57	(5.56)	13.31	(4.51)	4.06	66	<.001	*	PTSD>TC
Physical abuse (SD)	12.02	(6.51)	9.81	(3.84)	1.77	65.89	.082	†	PTSD(>)TC
Sexual abuse (SD)	16.27	(6.78)	10.27	(5.70)	3.76	66	<.001	*	PTSD>TC
Emotional neglect (SD)	19.12	(5.13)	12.88	(4.46)	5.12	66	<.001	*	PTSD>TC
Physical neglect (SD)	12.51	(4.84)	7.31	(2.65)	5.72	65.23	<.001	*	PTSD>TC
Davidson Trauma Scale (DTS)									
Total (SD)	77.73	(18.89)	12.04	(12.80)	15.35	64	<.001	*	PTSD>TC
Intensity (SD)	39.42	(10.97)	5.44	(6.43)	15.86	63.94	<.001	*	PTSD>TC
Frequency (SD)	38.32	(9.65)	6.73	(6.42)	14.74	65	<.001	*	PTSD>TC
Beck Depression Inventory 2 (BDI-II)									
Total (SD)	36.30	(11.66)	4.01	(5.35)	15.51	61.88	<.001	*	PTSD>TC
MACE									
Severity (SD)	22.92	(12.69)	8.83	(6.04)	6.160	62.717	<.001	*	PTSD>TC
Duration (SD)	13.79	(2.76)	11.23	(3.83)	2.958	41.110	.005	*	PTSD>TC
MULTI (SD)	6.83	(2.24)	4.69	(1.85)	4.083	66	<.001	*	PTSD>TC
MACE TRAUMA TYPES									
Neglect overall (SD)	7.03	(5.29)	2.10	(2.90)	4.94	65.26	<.001	*	PTSD>TC
Abuse overall (SD)	4.82	(3.55)	2.06	(1.60)	4.37	61.52	<.001	*	PTSD>TC
Emotional Abuse Parents (SD)	4.32	(2.32)	2.39	(2.12)	3.45	66	.001	*	PTSD>TC
Emotional Abuse Siblings (SD)	1.04	(1.63)	.14	(.43)	3.39	49.60	.001	*	PTSD>TC
Physical Abuse Parents (SD)	3.52	(2.95)	1.74	(1.55)	3.26	64.66	.002	*	PTSD>TC
Physical Abuse Siblings (SD)	.66	(1.50)	.10	(.38)	2.32	49.01	.025	*	PTSD>TC
Sexual Abuse (SD)	.64	(.62)	.23	(.34)	3.53	65.27	.001	*	PTSD>TC
Emotional Neglect (SD)	4.74	(3.50)	1.50	(2.30)	4.61	65.72	<.001	*	PTSD>TC

Physical Neglect (SD)	2.29	(2.24)	0.60	(.90)	4.34	58.68	<.001	*	PTSD>TC
Peer Abuse (SD)	1.91	(1.91)	0.66	(.73)	3.82	57.27	<.001	*	PTSD>TC
Witnessing Abuse between Parents (SD)	1.51	(2.03)	0.40	(.69)	3.23	54.73	.002	*	PTSD>TC
Witnessing Abuse towards Siblings (SD)	2.29	(2.23)	1.07	(1.34)	2.81	65.96	.006	*	PTSD>TC
Current Comorbidities N (%)									
Affective Disorder	27	(64.3)							
Substance Dependency	0	(0)							
Substance Abuse	1	(2.4)							
Anxiety Disorder	30	(71.4)							
Obsessive Compulsive Disorder	7	(16.7)							
Somatization Disorder	3	(7.1)							
Eating Disorder	5	(11.9)							
Borderline Personality Disorder	20	(47.6)							
Psychotropic Medication N (%)									
SSRI	11	(26.2)							
SNRI	10	(23.8)							
Tricyclica	5	(11.9)							
Other Antidepressants	4	(9.5)							
Neuroleptics	8	(19.1)							
Anticonvulsants	3	(7.1)							
Unmedicated	15	(35.7)							

Legend: CTQ abuse - total (SD) = CTQ physical abuse + CTQ sexual abuse; CTQ neglect - total (SD) = CTQ emotional neglect + CTQ physical neglect. MACE = Maltreatment and Abuse Chronology of Exposure scale, SD = standard deviation, severity = ACE severity averaged across age 3 up to 17 with respect of all ten types of ACE, duration = averaged years of traumatization reported across age 3 up to 17, types = average numbers of ACE types experienced between age 3 up to 17 (maximal value = 10)

2.3 Effects of ACE and PTSD on brain volume

2.3.1 Whole brain volume analysis

No significant differences in brain volume were observed.

2.3.2 Regional brain volume analysis

2.3.2.1 Amygdala: Groups did differ in their amygdala volume irrespective of the hemisphere (group: F(1,66) = 4.89, p = .030, group x hemisphere: F(1,66) < .01, p = .983, Figure S2 A): PTSD subjects had a smaller amygdala volume compared to TC subjects.

2.3.2.2 Hippocampus: Groups did not differ in hippocampal volume (group: F(1,66) = 1.77, p = .189, group x hemisphere: F(1,66) = .06, p = .816, Figure S2 B).

2.3.2.3 Anterior Cingulate Cortex: Groups did differ in their ACC volume depending on the hemisphere (group x hemisphere: F(1,66) = 4.65, p = .035; group: F(1,66) = 4.65, p = .035, Figure S2 C): PTSD subjects had a smaller right ACC volume compared to TC subjects (p = .035), while there was a trend towards a smaller left ACC volume in PTSD compared to TC subjects (p = .064).

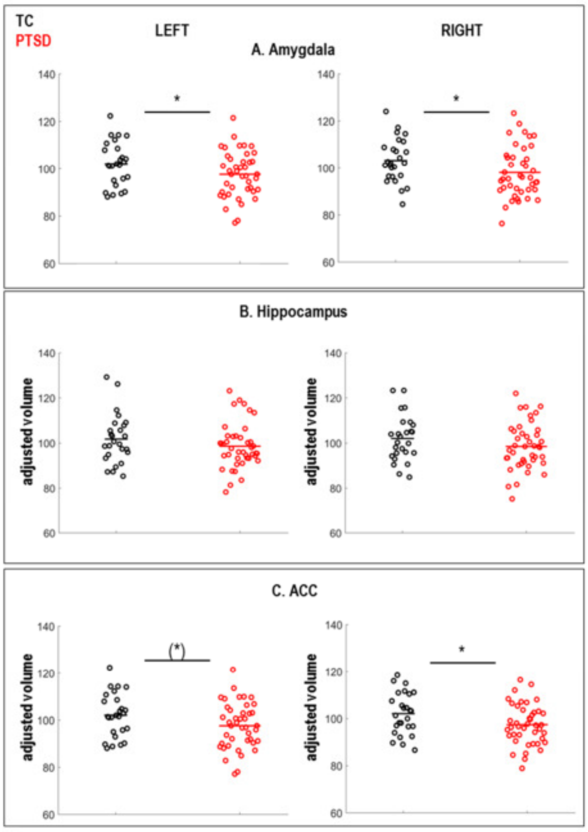

Figure S2. Differences in amygdala (A), hippocampus (B), and ACC (C) volume adjusted for age in PTSD, and TC subjects.

2.4 Importance of ACE Timing in Predicting Brain Volume

2.4.1 Anterior Cingulate Cortex

Sensitive period analyses revealed that *time-specific ACE severity* at 10 years of age was important in predicting left, while *time-specific ACE severity* at 3 years of age was important in predicting right ACC volume (for *p*-values of VI scores and trends see Table S6). With respect to global predictors, *global ACE severity* was found to be an important predictor for left ACC volume, while the predictor *group* was found to be important in predicting right ACC volume by trend (Table S6).

2.5 Importance of ACE Type in Combination with Timing in Predicting Brain Volume

2.5.1 Anterior Cingulate Cortex

Sensitive period analyses revealed that *time-specific abuse severity* at 7 years of age, and *time-specific neglect severity* at 3, and 4 years of age were important in predicting left, while *time-specific neglect severity* at 3, and 4 years of age were important in predicting right ACC volume (for *p*-values of VI scores and trends see Table S6). With respect to global predictors, *global abuse severity* was found to be an important predictor on a marginal significant level for left, and *global neglect severity* for right ACC volume, scores while the predictor *group* was found to be important in predicting right ACC volume only (Table S6).

2.6 Importance of the Severity of a specific ACE Type in Combination with Timing in Predicting Brain Volume

To exploratory investigate whether the observed importance of neglect in predicting amygdala and hippocampal volume during specific time periods was mainly related to the inclusion of the severity of abuse into the type x timing model, we additional run separate random forest regression analyses including either a) the severity of neglect during specific time periods, or b) the severity of abuse during specific time periods as predictor variables in predicting amygdala or hippocampal volume.

2.6.1 Abuse

2.6.1.1 Amygdala Volume

Sensitive period analyses revealed no *time-specific ACE severity* was important in predicting left, or right amygdala volume (for *p*-values of VI scores and trends see Table S9). With respect to global predictors, the predictor *group* was found to be important in predicting both left, and right amygdala volume (Table S9, Figure S3 A).

2.6.1.2 Hippocampus Volume

Sensitive period analyses revealed that *time-specific abuse severity* at age 16 was important in predicting left hippocampal volume (for *p*-values of VI scores and trends see Table S9). With respect to global predictors, the predictor *group* was found to be important in predicting right hippocampus volume (Table S9, Figure S3 B).

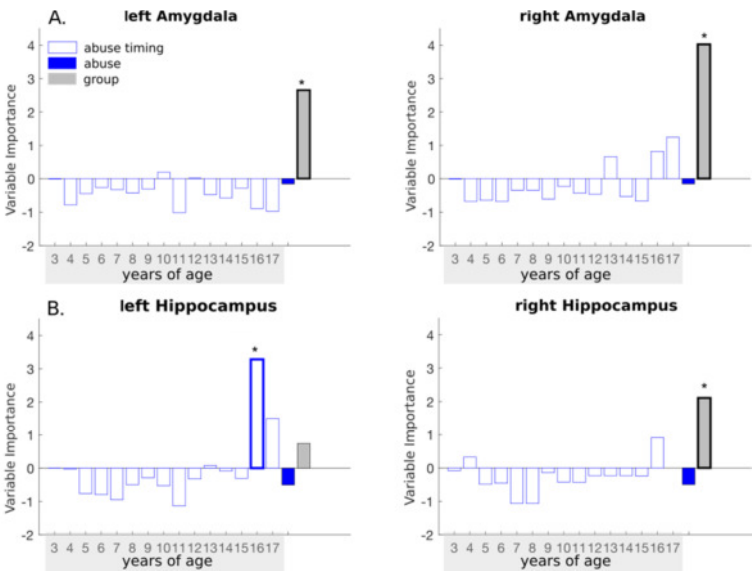

Figure S3. Results of random forest regression with conditional interference trees indicating the importance of time-specific abuse severity from 3 up to 17 years of age on bilateral amygdala (A.), and hippocampal volume (B.).
*permutation test: * p < .05; † < .1*

2.6.2 Neglect

2.6.2.1 Amygdala Volume

Sensitive period analyses revealed that *time-specific neglect severity* at age 11 was important in predicting right amygdala volume (for *p*-values of VI scores and trends see Table S9). With respect to global predictors, none of the latter were found to be important in predicting left or right amygdala volume (Table S9, Figure S4 A).

2.6.2.2 Hippocampus Volume

Sensitive period analyses revealed that *time-specific neglect severity* at age 10 was important in predicting right hippocampus volume (for *p*-values of VI scores and trends see Table S9). With respect to global predictors, none of the latter were found to be important in predicting left or right hippocampal volume (Table S9, Figure S4 B).

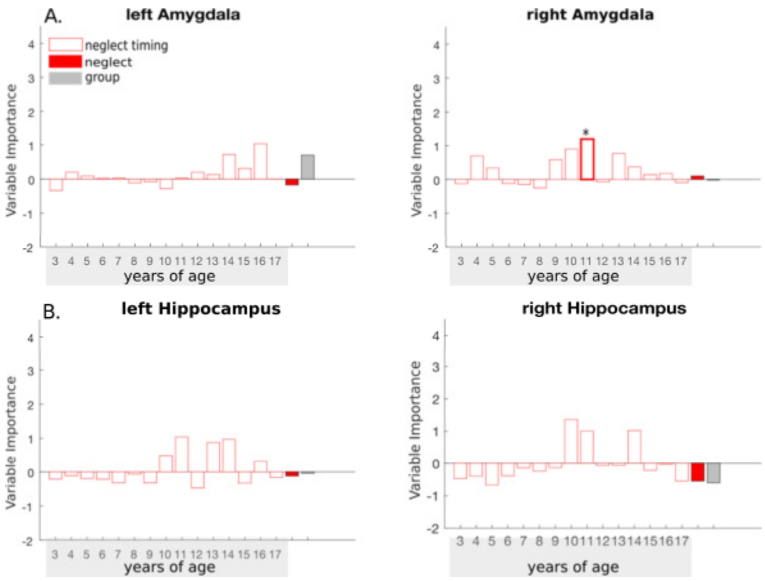

Figure S4. Results of random forest regression with conditional interference trees indicating the importance of time-specific neglect severity from 3 up to 17 years of age on bilateral amygdala (A.), and hippocampal volume (B.).
*permutation test: * p < .05; † < .1*

Table S6. Sensitive period analysis of ROI volume using random forest regression with conditional interference trees indicating significant of identified predictors and fit based on randomized resampling.

Region	Predictor	Peak VI[a]	*p*	
**Amygdala**				
left	13	2.74	.024	*
left	global severity	-0.56	.931	ns
left	group	0.93	.116	ns
right	10	1.54	.048	*
right	13	2.73	.015	*
right	global severity	-0.39	.791	ns
right	group	-0.19	.411	ns
**Hippocampus**				
left	9	1.17	.061	†
left	10	3.08	.008	*
left	11	3.15	.010	*
left	13	2.51	.021	*
left	global severity	0.66	.103	ns
left	group	-0.45	.621	ns
right	9	0.89	.087	†
right	10	1.67	.033	*
right	11	1.96	.025	*
right	13	2.26	.016	*
right	global severity	0.15	.308	ns
right	group	0.20	.204	ns
**ACC**				
left	4	1.28	.096	†
left	8	0.89	.084	†
left	9	0.76	.098	†
left	10	1.18	.049	*
left	global severity	1.14	.019	*

left	group	0.52	.169	ns
right	3	2.15	.039	*
right	4	1.37	.061	†
right	9	0.73	.094	†
right	10	0.94	.066	†
right	11	0.90	.066	†
right	global severity	0.34	.150	ns
right	group	1.43	.056	†

** p< .05, †< .1*
[a] Variable importance indicating the decrease in model accuracy

Table S7. Interaction between sensitive periods and ACE type, i.e. time-specific neglect severity and time-specific abuse severity, on ROI volume using random forest regression with conditional interference trees indicating significant of identified predictors based on randomized resampling.

Region		Predictor	Peak VI[a]	*p*	
**Amygdala**					
	left	N[d] 6	0.56	.063	†
	left	N[d] 12	0.46	.094	†
	left	N[d] 13	0.46	.090	†
	left	N[d] 14	0.98	.036	*
	left	N[d] 15	0.80	.059	†
	left	N[d] 16	1.15	.030	*
	left	N[d] 17	.039	.099	†
	left	neglect global	0.36	.110	ns
	left	abuse global	-.19	.662	ns
	left	group	0.56	.110	ns
	right	N[d] 4	1.29	.029	*
	right	N[d] 5	0.46	.089	†

	right	N^d 6	0.64	.049	*
	right	N^d 9	0.88	.041	*
	right	N^d 10	0.59	.071	†
	right	N^d 11	1.54	.006	*
	right	N^d 13	1.29	.021	*
	right	N^d 14	1.15	.014	*
	right	N^d 17	0.57	.074	†
	right	neglect global	1.14	.013	*
	right	abuse global	<0.01	.366	ns
	right	group	<0.01	.293	ns
**Hippocampus**					
	left	A^e 16	1.43	.030	*
	left	A^e 17	1.01	.040	*
	left	N^d 9	0.70	.044	*
	left	N^d 10	0.53	.085	†
	left	N^d 11	0.86	.037	*
	left	N^d 13	0.97	.028	*
	left	N^d 14	1.24	.017	*
	left	N^d 16	0.63	.064	†
	left	neglect global	0.21	.203	ns
	left	abuse global	0.22	.188	ns
	left	group	0.04	.249	ns
	right	N^d 10	0.72	.046	*
	right	N^d 11	1.27	.012	*
	right	N^d 13	0.75	.028	*
	right	N^d 14	0.67	.061	†
	right	N^d 15	0.47	.098	†
	right	neglect global	0.24	.165	ns
	right	abuse global	-0.48	.933	ns

right	group	0.01	.262	ns
**ACC**				
left	A^e 7	1.07	.041	*
left	A^e 8	0.78	.065	†
left	A^e 14	0.54	.090	†
left	N^d 3	0.88	.042	*
left	N^d 4	1.61	.009	*
left	N^d 5	0.41	.098	†
left	N^d 7	0.34	.095	†
left	neglect global	0.07	.311	ns
left	abuse global	0.59	.080	†
left	group	0.58	.109	ns
right	N^d 3	0.84	.031	*
right	N^d 4	1.19	.011	*
right	N^d 5	0.45	.070	†
right	N^d 8	0.35	.079	†
right	N^d 10	0.35	.088	†
right	neglect global	0.67	.027	*
right	abuse global	0.26	.146	ns
right	group	0.91	.049	*

**p< .05, †<.1*

[a] *Variable importance(VI) indicating the decrease in model accuracy*

Table S8. Results of generalized linear model regression for severity of type and timing related variables in predicting amygdala, hippocampus and ACC volume.

	**model**	**linear term**			**quadratic term**			**model comparison**	
		beta-value	***T-value***	***p***	***beta-value***	***T-value***	***p***	***F-value***	***p***
**left amygdala**									
**ACE 13**	**linear**	-.0001	-2.04	.045	--	--	--	.02	.885
	quadratic	-.0001	-.81	.422	<.001	.15	.884		
**Neglect 14**	**linear**	-.0003	-2.20	.031	--	--	--	.75	.390
	quadratic	-.0007	-1.49	.140	<.001	.87	.386		
**Neglect 16**	**linear**	-.0003	-2.30	.024	--	--	--	.19	.665
	quadratic	-.0005	-1.13	.261	<.001	.44	.663		
**right amygdala**									
**ACE 10**	**linear**	-.0001	-2.02	.048	--	--	--	1.19	.279
	quadratic	.0001	0.39	.696	<.001	-1.09	.276		
**ACE 13**	**linear**	-.0001	-1.92	.058	--	--	--	.002	.964
	quadratic	-.0001	-.67	.504	<.001	.05	.963		
**Neglect 4**	**linear**	-.0005	-2.58	.012	--	--	--	<.001	.990
	quadratic	-.0005	-0.97	.335	<.001	0.01	.990		
**Neglect 6**	**linear**	-.0004	-2.31	.024	--	--	--	.72	.399
	quadratic	-.0008	-1.58	.118	<.001	0.86	.395		
**Neglect 9**	**linear**	-.0004	-2.60	.011	--	--	--	.45	.505
	quadratic	-.0007	-1.49	.139	<.001	0.68	.502		
**Neglect 11**	**linear**	-.0004	-2.68	.009	--	--	--	.77	.383
	quadratic	-.0008	-1.69	.096	<.001	.89	.379		
**Neglect 13**	**linear**	-.0004	-2.62	.011	--	--	--	.40	.527
	quadratic	-.0007	-1.42	.160	<.001	.64	.524		
**Neglect 14**	**linear**	-.0004	-2.52	.014	--	--	--	.25	.616
	quadratic	-.0006	-1.24	.219	<.001	.51	.613		

**left hippocampus**									
**ACE 10**	**linear**	-.0003	-1.69	.096	--	--	--	4.33	.041
	quadratic	.0007	1.43	.158	<.001	-2.09	.039		
**ACE 11**	linear	-.0002	-1.49	.139	--	--	--	2.75	.102
	quadratic	.0005	1.07	.289	<.001	-1.67	.099		
**ACE 13**	linear	-.0002	-1.56	.124	--	--	--	1.04	.312
	quadratic	.0002	.46	.650	<.001	-1.03	.307		
**Neglect 9**	**linear**	-.0007	-1.74	.087	--	--	--	.005	.939
	quadratic	-.0006	-.49	.622	<.001	-.08	.939		
**Neglect 11**	**linear**	-.0008	-2.68	.009	--	--	--	.77	.383
	quadratic	-.0016	-1.69	.096	<.001	.89	.379		
**Neglect 13**	**linear**	-.0009	-2.24	.029	--	--	--	.08	.775
	quadratic	-.0012	-.97	.338	<.001	.29	.773		
**Neglect 14**	**linear**	-.0008	-1.97	.053	--	--	--	.03	.866
	quadratic	-.0011	-.75	.455	<.001	.17	.865		
**Abuse 16**	**linear**	.0017	2.51	.015	--	--	--	.32	.577
	quadratic	.0027	1.37	.176	-.0001	-.57	.574		
**Abuse 17**	**linear**	.0013	1.78	.079	--	--	--	.02	.902
	quadratic	-.0008	.38	.706	<.001	.12	.901		
**right hippocampus**									
**ACE 10**	**linear**	-.0003	-1.94	.057	--	--	--	1.68	.199
	quadratic	.0003	.61	.544	<.001	-1.31	.196		
**ACE 11**	**linear**	-.0002	-1.74	.087	--	--	--	.54	.465
	quadratic	.0001	.12	.907	<.001	.74	.462		
**ACE 13**	**linear**	-.0003	-1.75	.085	--	--	--	.01	.911
	quadratic	.0002	-.47	.642	<.001	-.11	.911		
**Neglect 10**	**linear**	-.0008	-2.04	.046	--	--	--	.62	.434

	quadratic	-.0016	-1.43	.159	.0001	.73	.430		
**Neglect 11**	**linear**	-.0008	-2.02	.047	--	--	--	.99	.323
	quadratic	-.0019	-1.59	.116	.0001	1.00	.319		
**Neglect 13**	**linear**	-.0008	-2.18	.033	--	--	--	.19	.666
	quadratic	-.0013	-1.09	.281	<.001	.44	.663		
**left ACC**									
**ACE 10**	linear	<.001	-.35	.725	--	--	--	3.25	.076
	quadratic	.0015	1.61	.113	<.001	-1.81	.074		
**Neglect 3**	**linear**	-.0017	-1.69	.095	--	--	--	.99	.325
	quadratic	<.001	.30	.764	<.001	-1.00	.321		
**Neglect 4**	**linear**	-.0018	-1.77	.081	--	--	--	.94	.335
	quadratic	<.001	.24	.809	<.001	-.98	.331		
**Abuse 7**	linear	.0007	.67	.506	--	--	--	.479	.491
	quadratic	-.0010	-.37	.711	.0001	.69	.488		
**right ACC**									
**ACE 3**	linear	-.1724	-1.40	.166	--	--	--	.002	.964
	quadratic	-.1557	-.41	.685	-.0007	-.05	.963		
**Neglect 3**	**linear**	-.4244	-1.81	.074	--	--	--	1.38	.245
	quadratic	.2685	.42	.672	-.0512	-1.18	.241		
**Neglect 4**	linear	-.3749	-1.61	.113	--	--	--	1.85	.178
	quadratic	.4117	.66	.508	-.0585	-1.34	.174		

** p<.05, †<.1.*
bold notation highlight favoured model

Table S9. Sensitive period analysis and severity of ACE type, respectively, i.e. time-specific neglect severity and time-specific abuse severity, on ROI volume using random forest regression with conditional interference trees indicating significant of identified predictors based on randomized resampling.

Region	Predictor	Peak VI[a]	*p*	
**Amygdala**				
abuse				
left	group	2.65	.041	*
right	17	1.25	.073	†
right	group	4.02	.019	*
neglect				
left	16	1.04	.069	†
right	10	.90	.085	†
right	11	1.19	.049	*
**Hippocampus**				
abuse				
left	16	3.28	.020	*
left	17	1.50	.054	†
right	group	2.11	.045	*
- neglect				
left	11	1.03	.057	†
left	13	.87	.090	†
left	14	.96	.072	†
right	10	1.36	.046	*
right	11	1.01	.064	†
right	14	1.02	.070	†

* *p<.05, †<.1*

[a]*Variable importance(VI) indicating the decrease in model accuracy,*

References

1. Wittchen H-U, Wunderlich U, Gruschwitz S, Zaudig M-. *SKID I Strukturiertes Klinisches Interview für DSM-IV Achse I: Psychische Störungen Interviewheft und Beurteilungsheft Eine deutschsprachige, erweiterte Bearbeitung der amerikanischen Originalversion des SCID I.* Göttingen: Hogrefe; 1997.
2. The clinician-administered PTSD scale for DSM-5 (CAPS-5). Interview available from the National Center for PTSD. 2013.
3. Loranger AW, Janca A, Sartorius N. *Assessment and diagnosis of personality disorders: The ICD-10 international personality disorder examination (IPDE).* New York, NY: Cambridge University Press; 1997.
4. Bernstein DP, Fink L. *Childhood Trauma Questionnaire: A retrospective self-report manual* San Antonio: TX: The Psychological Corporation; 1998.
5. Davidson JR, Book SW, Colket JT, et al. Assessment of a new self-rating scale for post-traumatic stress disorder. *Psychological Medicine.* 1997;27(1):153-160.
6. Hautzinger M, Kuehner C, Buerger C, Keller F. *Manual für das BDI-II.* Bern: Huber; 2003.
7. Rausch S, Herzog J, Thome J, et al. Women with exposure to childhood interpersonal violence without psychiatric diagnoses show no signs of impairment in general functioning, quality of life and sexuality. *Borderline Personal Disord Emot Dysregul.* 2016;3:13.
8. Teicher MH, Parigger A. The 'Maltreatment and Abuse Chronology of Exposure' (MACE) scale for the retrospective assessment of abuse and neglect during development. *PloS one.* 2015;10(2):e0117423.
9. Isele D, Teicher MH, Ruf-Leuschner M, et al. KERF–An instrument for measuring adverse childhood experiences: Construction and psychometric evaluation of the German MACE (Maltreatment and Abuse Chronology of Exposure) scale. *Z Klin Psych Psychoth.* 2014;43:121–130.
10. McLaughlin KA, Sheridan MA, Lambert HK. Childhood adversity and neural development: deprivation and threat as distinct dimensions of early experience. *Neuroscience and biobehavioral reviews.* 2014;47:578-591.
11. Sheridan MA, McLaughlin KA. Dimensions of early experience and neural development: deprivation and threat. *Trends in cognitive sciences.* 2014;18(11):580-585.
12. Strobl C, Boulesteix AL, Kneib T, Augustin T, Zeileis A. Conditional variable importance for random forests. *BMC bioinformatics.* 2008;9:307.
13. Strobl C, Malley J, Tutz G. An introduction to recursive partitioning: rationale, application, and characteristics of classification and regression trees, bagging, and random forests. *Psychological methods.* 2009;14(4):323-348.
14. Kuhn M, Johnson K. *Applied predictive modeling.* New York: Springer; 2016.
15. Breiman L. Random forests. *Machine learning.* 2001;45(1).
16. Pechtel P, Lyons-Ruth K, Anderson CM, Teicher MH. Sensitive periods of amygdala development: the role of maltreatment in preadolescence. *Neuroimage.* 2014;97:236-244.
17. Altmann A, Tolosi L, Sander O, Lengauer T. Permutation importance: a corrected feature importance measure. *Bioinformatics (Oxford, England).* 2010;26(10):1340-1347.
18. Janitza S, Celik E, Boulesteix A-L. A computationally fast variable importance test for random forests for high-dimensional data. *Advances in Data Analysis and Classification.* 2016.
19. Durstewitz D. *Advanced Data Analysis in Neuroscience: Integrating Statistical and Computational Models.* Springer International Publishing; 2017.
